# Socio-demographic and health-related determinants of patients’ overall rating and experiences of cancer care

**DOI:** 10.1186/s12885-023-11445-6

**Published:** 2023-09-29

**Authors:** Chantal Arditi, Manuela Eicher, Julien Junod, Isabelle Peytremann-Bridevaux

**Affiliations:** 1 Department of Epidemiology and Health Systems, Center for Primary Care and Public Health (Unisanté), University of Lausanne, Lausanne, Switzerland; 2 Institute of Higher Education and Research in Healthcare (IUFRS), Faculty of Biology and Medicine, University of Lausanne, Lausanne, Switzerland; 3 Department of Oncology, Lausanne University Hospital (CHUV), Lausanne, Switzerland

**Keywords:** Cancer, Patient survey, Patient experiences, Patient satisfaction, Quality of care, Switzerland

## Abstract

**Background:**

Understanding how patient-reported experiences of care and overall rating of care vary among patients with different characteristics is useful to help interpret results from patient experience surveys and design targeted improvement interventions. The primary objective of this paper was to identify the socio-demographic and health-related characteristics independently associated with overall rating of cancer care. The secondary objective was to explore if and how these characteristics were associated with specific experiences of cancer care.

**Methods:**

This cross-sectional multicenter study analyzed self-reported data collected from 2696 patients diagnosed with breast, prostate, lung, colorectal, skin, or hematological cancer from four large hospitals in French-speaking Switzerland. Multivariate logistic regressions with purposeful stepwise selection of independent variables were used to identify the socio-demographic and health-related characteristics independently associated with overall rating of cancer care in the primary analyses. In the secondary analyses, we ran the multivariate model from the primary analyses with specific experiences of care as outcomes to estimate the adjusted odds ratios (OR) and 95% confidence intervals (CI) of the selected characteristics.

**Results:**

Respondents’ mean rating of overall cancer care was 8.5 on a scale from 0 to 10, with 17% categorized as reporting a low rating (0–7 rating). Being a woman (OR 1.43, 95% CI 1.12–1.83), not being Swiss (OR 1.47, 95% CI 1.12–1.94), reporting lower health literacy (OR 1.95, 95% CI 1.54–2.47), preferring making medical decisions alone (OR 1.92, 95% CI 1.38–2.67), having forgone care due to cost (OR 1.72, 95% CI 1.29–2.29), having used complementary medicine (OR 1.55, 95% CI 1.22–1.97), and reporting poorer health (OR 3.12, 95% CI 2.17–4.50) were all independently associated with a low rating of overall cancer care. Poorer health, lower health literacy, and having forgone care were the three characteristics most often associated with problematic experiences of care.

**Conclusions:**

Our results identified several patient characteristics consistently associated with lower overall rating of care and specific experiences of cancer care. Among these determinants, health literacy and financial hardship emerged as key recurring factors shaping poor patient experiences that should be prioritized for attention by cancer care services.

**Supplementary Information:**

The online version contains supplementary material available at 10.1186/s12885-023-11445-6.

## Background

Patients-reported experience measures (PREMs) are now widely recognized as one of the important quality indicators of cancer care, along other indicators such as clinical and mortality outcomes. These PREMs are typically collected with online and/or paper patient surveys, asking patients to report on their experiences while receiving cancer care and interacting with health professionals. Cancer PREMs surveys usually include a number of items asking about specific experiences of care along the cancer care continuum, spanning from detection and diagnosis to follow-up care, and often end with an overall rating of care, used as an aggregated measure of overall experience [1, 2]. Understanding how overall rating and specific experiences of care vary among patients with different characteristics is useful to help interpret results from patient surveys and to design targeted improvement intervention. Previous studies and a recent systematic review [3] have shown that cancer patients’ experiences vary quite significantly by a wide array of patients’ characteristics, sometimes in an inconsistent manner. Regarding socio-demographic characteristics, older age was reported to be either positively [4–8], negatively [9] or not related to positive ratings of care [10, 11]. Being married was associated with lower ratings in one study [11] while being single was associated with lower ratings in another study [12]. There was more consensus around women tending to report less positively [4, 6–8, 11, 13, 14], as patients from ethnic minorities [7, 8, 11, 14, 15] and lower income [11, 13, 15]. Other frequent socio-demographic determinants of patient experiences reported in the literature were area of residence [5, 13, 16], education level [5, 6, 10], and level of social support [6, 11]. Regarding health characteristics, poorer health status or quality of life was one of the most important determinants of reporting lower ratings and poorer experiences [5, 6, 9, 10, 12, 15, 17–19]. Finally, studies looking at clinical characteristics of cancer have found that experiences varied by type of cancer and prognosis [4, 6, 9, 13, 16], treatments [8, 9] and time since diagnosis [12]. To date, there were no studies in Switzerland examining the interplay between patient-related characteristics and overall rating of cancer care and specific experiences of care. As the Swiss healthcare system differs from the UK and the USA systems where most previous research was done, one might expect different determinants than in other countries. In addition, we did not find studies examining health literacy as a determinant of patient experiences, despite low literacy being a predictor of inadequate use of health care services and poor health outcomes [25, 26]. Our primary objective was to identify the socio-economic and health-related characteristics independently associated with the overall rating of cancer care in Switzerland. The secondary objective was to explore if and how these characteristics were associated with specific experiences of care.

## Methods

### Study design and population

We used data collected for the Swiss Cancer Patient Experiences (SCAPE) study, an observational cross-sectional multicenter survey of patients diagnosed with cancer in four large hospitals in the French-speaking region of Switzerland [20]. Patient eligibility criteria were adult Swiss residents (≥ 18 years) with a confirmed diagnosis of breast, prostate, lung, colorectal, skin, or hematological cancer (leukemia, lymphoma, myeloma); who were hospitalized or had an outpatient visit in an oncology unit at the recruiting hospital; between January 1, 2018 and June 30, 2018. Data were collected with a questionnaire mailed to eligible patients in October 2018. The SCAPE questionnaire was adapted from the 2016 version of the questionnaire used annually in the United Kingdom [21], which was translated into French and culturally adapted following international guidelines [22, 23]. Patients could complete and return the survey questionnaire by post or complete it online. Non-respondents received a reminder in January 2019. We included in the analyses individuals reporting an eligible cancer who returned the questionnaire by the end of March 2019.

### Overall rating of cancer care (primary outcome of interest)

**Fig. 1:**
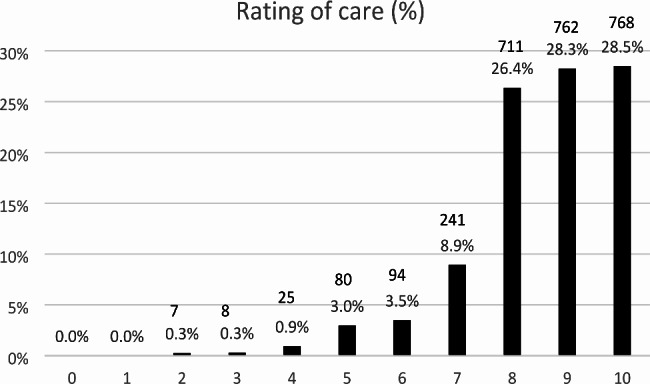
Frequency (%) of ratings of overall cancer care

Overall rating of cancer care was measured with the following question, at the end of the first section of the self-administered questionnaire about experiences of care: ‘How would you rate your overall cancer care?’, with a 0 (worst) to 10 (best) rating scale. We dichotomized answers as ‘high’ if rating was between 8 and 10 and ‘low’ if below 8 based on the step shape of the response distribution (see Fig. 1). We excluded from the analyses patients who did not answer the question (n = 59).

### Specific experiences of care (secondary outcomes of interest)

Outcomes of interest for the secondary objective comprised what we defined as ‘general’ and ‘recent’ experiences of care. ‘General’ experiences pertained to experience questions regarding cancer care in general that applied to all patients, while ‘recent’ experiences came from experience questions asking patients to answer only if they had had the targeted health care service within the last 12 months. The ‘general’ experiences of care could have happened more than 12 months before the survey, and included a large period of time, thus assessing overall cancer care. The ‘general’ experiences included 21 questions asking patients to evaluate: 1) waiting time before seeing an oncologist for the diagnosis; 2) the diagnosis process (told they could be accompanied, told in a sensitive manner, understood explanations, received written information); 3) treatment decision making (treatment options were explained, possible side-effects were explained, was offered support for dealing with side effects, told about long-term side effects, involved in treatment decisions; 4) home care and support (family given information to help care at home, care from health or social services during or after treatment; 5) support for people with cancer (received information on support groups, on impact of cancer on daily activities, on getting financial help; 6) care from the general practitioner (GP) (GP receiving information, GP support) and 7) overall aspects of care (professionals working well together, receiving a care plan and administration of care). The ‘recent’ experiences of care comprised 17 questions on: 1) diagnostic tests in the last 12 months (received information before, waiting time before, received explanations after; 2) inpatient hospital care in the last 12 months (staff talking while ignoring patient, trust in doctors, opportunity for family to talk to doctors, trust in nurses, enough nurses, enough privacy, found someone to talk about worries, received help with pain, treated with respect, received information about post discharge, told whom to contact if worried; and 3) outpatient hospital care in the last 12 months (found someone to talk about worries, doctors had documents available, waiting time). Most experience questions had a 5-point Likert-type scale response options to having a positive experience (i.e., “yes, completely”; “yes, to some extent”; “no”; “not applicable”; and “don’t know/can’t remember”). Responses were dichotomized as problematic (i.e. non-positive) experience, pooling “yes, to some extent” and ”no” answers, and non-problematic (i.e. positive or neutral) experience, pooling “yes, completely”, “don’t know/can’t remember”, and “not applicable” answers).

### Patient characteristics (explanatory variables)

Socio-demographic explanatory variables available were sex, age, marital status (single, married/partnership, divorced/separated, widowed), education (primary, secondary, tertiary), professional activity status (active, on disability or sick leave, retired), principal language (French, other), nationality (Swiss, non-Swiss), financial hardship (trouble paying household bills in past 12 months, forwent care due to costs in past 12 months), health literacy (frequency of having difficulty understating written medical information, a single screening question on functional health literacy shown to have good sensitivity and specificity to detect people with health literacy limitations and validated in French and in Switzerland [24, 25]) and medical decision making preference (with doctor, alone, doctor alone). Cancer and health-related explanatory variables were cancer diagnosis and treatments, use of complementary medicine, any comorbidity (list of 12 chronic conditions), overall health status, validated screening question of depressive symptoms [26], and a previously validated measure of health-related quality of life Functional Assessment of Cancer Therapy – General 7 item version (FACT-G7) [27]. Further details on the questionnaire, as well as the distribution of responses for patient characteristics and experiences of care, can be found in the previous publication on the analyses of the variation of reported experiences of care by type of cancer [20].

### Data analyses

For the primary analyses, we first ran descriptive analyses on patient characteristics (i.e., socio-demographic, health-related) and the overall rating of cancer care. We then performed univariate logistic regressions to identify explanatory variables associated with reporting a low rating of cancer care. To build a final multivariate model of factors independently associated with a low rating of cancer care, we started with all the variables associated with the outcome variable with a P-value of 0.15 or lower in the univariate logistic regressions. We then followed the ‘purposeful selection’ process suggested by Hosmer and colleagues [28] for the selection of explanatory variables. It corresponds to a backwards stepwise construction, in which one monitors the influence variables may exercise on each other. The role of each covariate was carefully assessed, detecting potential collinearities among covariates, and giving each previously eliminated covariate another chance to enter the model again. We used graphical representations to assess the influence of single or groups of observations on the model (leverage points) and to check whether subgroups (e.g. hospitals) were appropriately fitted by the model [29]. We used the likelihood ratio as goodness-of-fit measure. We reported unadjusted and adjusted odds ratio (OR) and 95% confidence intervals (CI) for the explanatory variables. We also performed sensitivity analyses by running the primary analyses with a low rating of care defined as a rating between 0 and 8 (instead of between 0 and 7).

For the secondary analyses, we ran the selected multivariate model from the primary analyses with the 21 general and 17 recent experiences of care as outcomes to estimate the adjusted OR and 95% CI of the selected characteristics, presented in forest plots for each characteristic. Each experience was analyzed separately as there are no validated dimensions in the questionnaire. All p-values were corrected for multiple testing using the False Discovery Rate method [30] in the secondary analyses, and we indicated in the plots if the p-value was under 0.05 after correction.

We estimated the intraclass correlation coefficients of mixed-models with a random effect for each hospital. As they were all below 0.01, the effect of hospital clustering was negligible and multilevel modelling not necessary. Missing data were not computed; all statistical analyses were conducted with Stata 16.1.

### Patient involvement

A Patient Partner took part in the study steering committee. She was also involved in pre-testing the questionnaire, writing the patient materials, answering patients’ email inquiries, analyzing the free-text comments, preparing and writing the lay results for patients, and disseminating the study and results on social media and to the scientific community.

## Results

### Participants’ characteristics

Of the 7145 patients invited to complete the survey, 3121 returned the questionnaire (44% participation rate). Of these, 2696 participants reported an eligible cancer and answered the question on the overall rating of care. Table 1 provides a descriptive overview of respondents’ characteristics. Their mean age was 63.8 and 61% were women. A third reported tertiary-level education, while a fifth reporting trouble paying household bills in the previous year. 81% of respondents reported a first cancer and 28% initiated their treatment within the previous year. The most frequently reported cancer was breast cancer (40%), followed by hematologic cancers (16%), lung cancer (15%), colorectal cancer (10%), prostate cancer (9%) and skin cancer (5%). A quarter reported excellent or very good health and mean quality of life was 19.3 on a scale from 0 to 28 (highest quality of life).

**Table 1:** Crude and adjusted odds ratios of a low rating of overall cancer care by socio-demographic and health-related characteristics

Variable	**N (%)**	**Low rating of overall cancer care**	Crude OR	Adjusted OR*
		**n (%)**	(95% CI)	(95% CI)
**Socio-demographic characteristics**				
Sex			p < 0.001	p = 0.004
Men	1046 (39.0)	140 (13.4)	1	1
Women	1634 (61.0)	313 (19.2)	1.53 (1.22–1.92)	1.43 (1.12–1.83)
Age			p = 0.001	-
18–54	591 (22.5)	127 (21.5)	1	
55–64	668 (25.4)	119 (17.8)	0.79 (0.60–1.05)	
65–74	848 (32.2)	126 (14.9)	0.64 (0.49–0.84)	
75+	524 (19.9)	72 (13.7)	0.58 0.42–0.80)	
Marital status			p = 0.010	-
Married/partnership	1598 (59.8)	240 (15.0)	1	
Separated/divorced	510 (19.1)	97 (19.0)	1.33 (1.02–1.72)	
Single	274 (10.3)	61 (22.3)	1.62 (1.18–2.22)	
Widowed	289 (10.8)	51 (17.7)	1.21 (0.87–1.69)	
Living situation			p = 0.013	-
Living with adult partner	1770 (66.2)	271 (15.3)	1	
Living without adult partner	767 (28.7)	150 (19.6)	1.34 (1.08–1.68)	
Other living arrangements	138 (5.2)	29 (21.0)	1.47 (0.16–0.21)	
Education			p = 0.264	-
Primary	418 (15.9)	80 (19.1)	1	
Secondary	1314 (50.0)	207 (15.8)	0.79 (0.59–1.05)	
Tertiary	898 (34.1)	152 (16.9)	0.86 (0.64–1.16)	
Professional activity status			p < 0.001	-
Active	729 (27.4)	126 (17.3)	1	
Disability or sick leave	287 (10.8)	62 (21.6)	1.32 (0.94–1.85)	
Retired	1395 (52.5)	199 (14.3)	0.80 (0.62–1.02)	
Other	248 (9.3)	57 (23.0)	1.43 (1.00-2.03)	
Principal language			p = 0.011	-
French	2312 (86.3)	374 (16.2)	1	
Other	367 (13.7)	79 (21.5)	1.42 (1.02–1.87)	
Nationality			p < 0.001	p = 0.006
Swiss	2230 (83.3)	348 (15.6)	1	1
Non-Swiss	446 (16.7)	105 (23.5)	1.67 (1.30–2.13)	1.47 (1.12–1.94)
Health literacy			p < 0.001	p < 0.001
High	1905 (72.3)	254 (13.3)	1	1
Low	731 (27.7)	191 (26.1)	2.30 (1.86–2.84)	1.95 (1.54–2.47)
Preference for making medical decisions			p < 0.001	p < 0.001
With doctor	2239 (84.3)	357 (15.9)	1	1
Alone	249 (9.4)	67 (26.9)	1.94 (1.43–2.63)	1.92 (1.38–2.67)
Doctor	169 (6.4)	23 (13.6)	0.83 (0.53–1.31)	0.73 (0.44–1.20)
Had trouble paying household bills			p < 0.001	-
No	2091 (78.9)	304 (14.5)	1	
Yes	559 (21.1)	136 (24.3)	1.89 (1.50–2.37)	
Forwent care due to costs			p < 0.001	p < 0.001
No	2294 (86.6)	339 (14.8)	1	1
Yes	354 (13.4)	102 (28.8)	2.33 (1.80–3.02)	1.72 (1.29–2.29)
**Health-related characteristics**				
Type of cancer			p = 0.008	-
Breast	1084 (40.2)	220 (20.3)	1	
Hematological	432 (16.0)	67 (15.5)	0.72 (0.53–0.97)	
Lung	405 (15.0)	58 (14.3)	0.66 (0.48–0.90)	
Colorectal	281 (10.4)	44 (15.7)	0.73 (0.51–1.04)	
Prostate	230 (8.5)	34 (14.8)	0.68 (0.46–1.01)	
Melanoma	138 (5.1)	14 (10.1)	0.44 (0.25–0.79)	
Several	126 (4.7)	18 (14.3)	0.65 (0.39–1.10)	
Type of diagnosis			p = 0.561	-
First cancer	2127 (80.5)	369 (17.4)	1	
Recurrence	271 (10.3)	42 (15.5)	0.87 (0.62–1.24)	
2nd or 3rd cancer	243 (9.2)	37 (15.2)	0.86 (0.59–1.24)	
Time since first treatment			p = 0.937	-
< 1 year	729 (27.7)	119 (16.3)	1	
1–5 years	1260 (47.9)	215 (17.1)	1.05 (0.83–1.35)	
> 5 years	640 (24.3)	112 (17.5)	1.09 (0.82–1.44)	
Treatment(s) received			p = 0.219	-
Surgery	1626 (28.6)	280 (17.2)	1.06 (0.86–1.30)	
Chemotherapy	1550 (27.3)	251 (16.2)	0.89 (0.72–1.09)	
Radiotherapy	1400 (24.7)	254 (18.1)	1.20 (0.98–1.48)	
Hormonotherapy	748 (13.2)	144 (19.3)	1.25 (1.01–1.56)	
Immunotherapy	352 (6.2)	55 (15.6)	0.90 (066-1.22)	
Use of complementary medicine			p < 0.001	p < 0.001
No	1782 (69.4)	262 (14.7)	1	1
Yes	785 (30.6)	173 (22.0)	1.64 (1.32–2.03)	1.55 (1.22–1.97)
Chronic comorbidities			p = 0.010	-
None	1067 (40.7)	155 (14.5)	1	
≥ 1 other than cancer	1553 (59.3)	285 (18.4)	1.32 (1.07–1.64)	
Overall health status			p < 0.001	p < 0.001
Excellent / Very good	660 (25.0)	59 (8.9)	1	1
Good	1532 (57.9)	260 (17.0)	2.08 (1.54–2.81)	1.77 (1.29–2.42)
Poor/bad	453 (17.1)	129 (28.5)	4.06 (2.90–5.68)	3.12 (2.17–4.50)
Depressive symptoms			p < 0.001	-
No	1678 (63.1)	223 (13.3)	1	
Yes	980 (36.9)	227 (23.2)	1.97 (1.60–2.41)	
Quality of life (0–28 highest)			p < 0.001	-
23–28	653 (24.7)	62 (9.5)	1	
20–22	700 (26.5)	95 (13.6)	1.47 (1.58–3.07)	
17–19	592 (22.4)	111 (18.8)	2.20 (0.33–0.63)	
0–16	701 (26.5)	184 (26.3)	3.39 (2.49–4.63)	

*Adjusted odds ratios from the multivariate model with the seven variables

### Overall rating of care and patient characteristics

Overall cancer care was rated at a mean 8.5 (standard deviation 1.4), with 17% of respondents categorized as reporting a low rating (0–7 ratings) (see Fig. 1).

The associations between patient characteristics and a low rating of overall care are shown in Table 1. Of the 21 patient characteristics under consideration, 17 were associated with overall rating of care in univariate analyses and seven remained in the final multivariate model (likelihood ratio 171.46; p-value < 0.001; pseudo R^2^ 0.08 indicating good fit). Being a woman (OR 1.43, 95% CI 1.12–1.83), not being Swiss (OR 1.47, 95% CI 1.12–1.94), reporting low health literacy (OR 1.95, 95% CI 1.54–2.47), preferring making medical decisions alone (OR 1.92, 95% CI 1.38–2.67), having forgone care due to cost (OR 1.72, 95% CI 1.29–2.29), having used complementary medicine (OR 1.55, 95% CI 1.22–1.97), and reporting poorer health (OR 3.12, 95% CI 2.17–4.50) were all independently associated with a higher likelihood of reporting a low rating of cancer care. Sensitivity analyses on the primary outcome with a cut-off at 8 for a low rating of care gave similar results (see Suppl. Table 1).

**Fig. 2:**
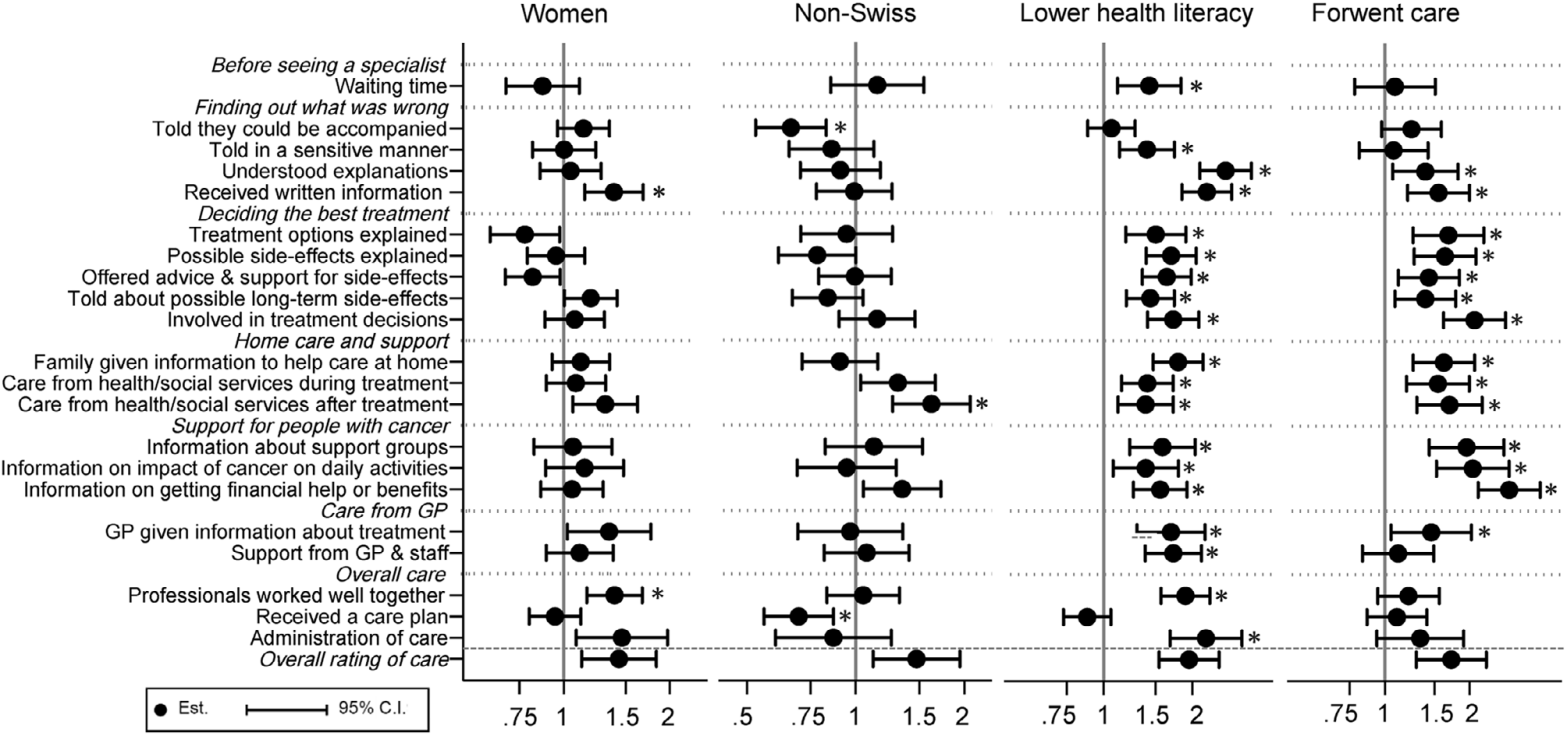
Adjusted OR and 95% CI of the patient characteristics (sex, nationality, health literacy, forgoing care) for reporting negative general experiences of care. * p-value < 0.05 after correction for multiple testing

**Fig. 3:**
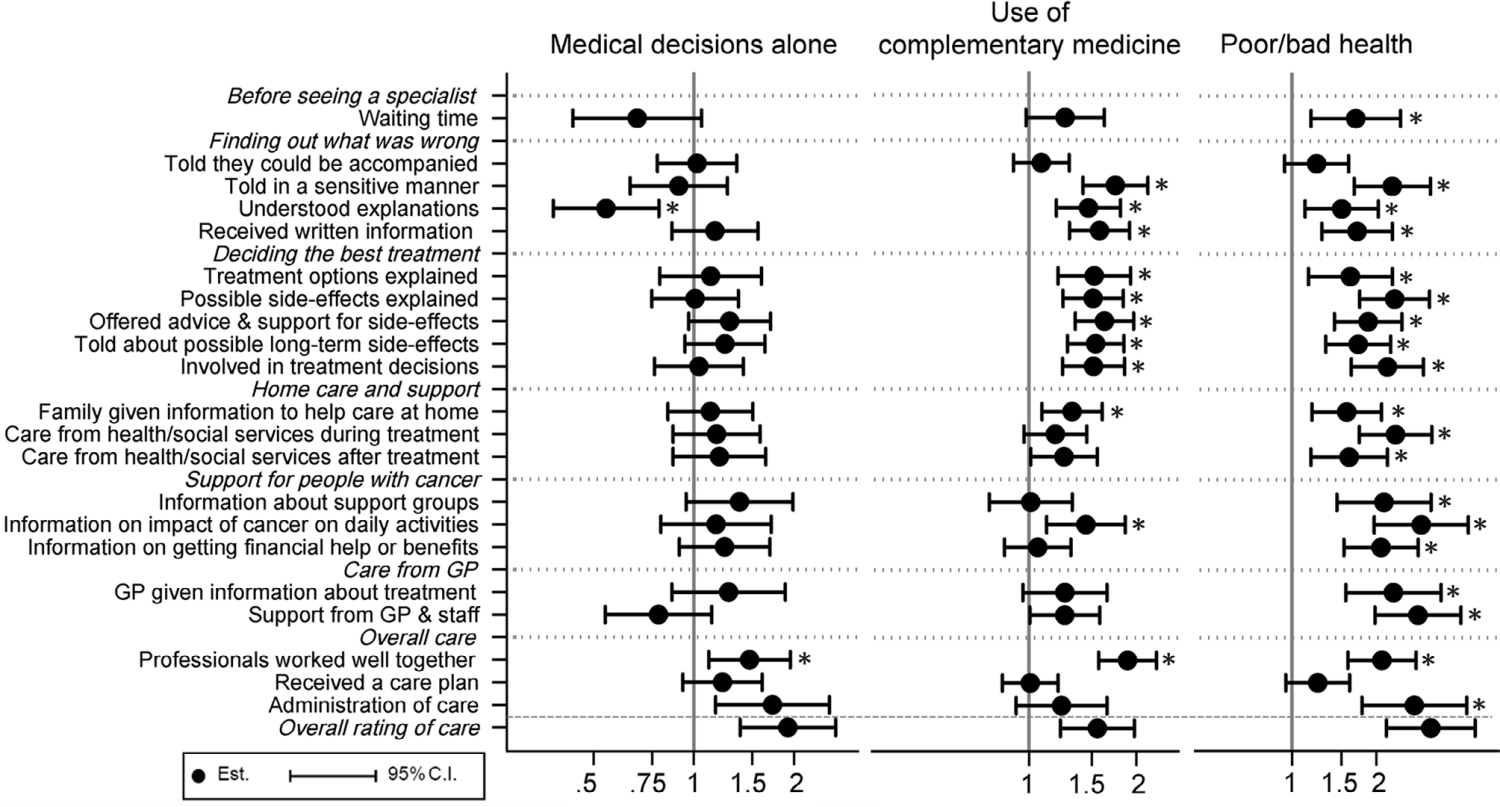
Adjusted OR and 95% CI of the patient characteristics (medical decision, complementary medicine, self-reported health) for reporting negative general experiences of care. * p-value < 0.05 after correction for multiple testing

**Fig. 4:**
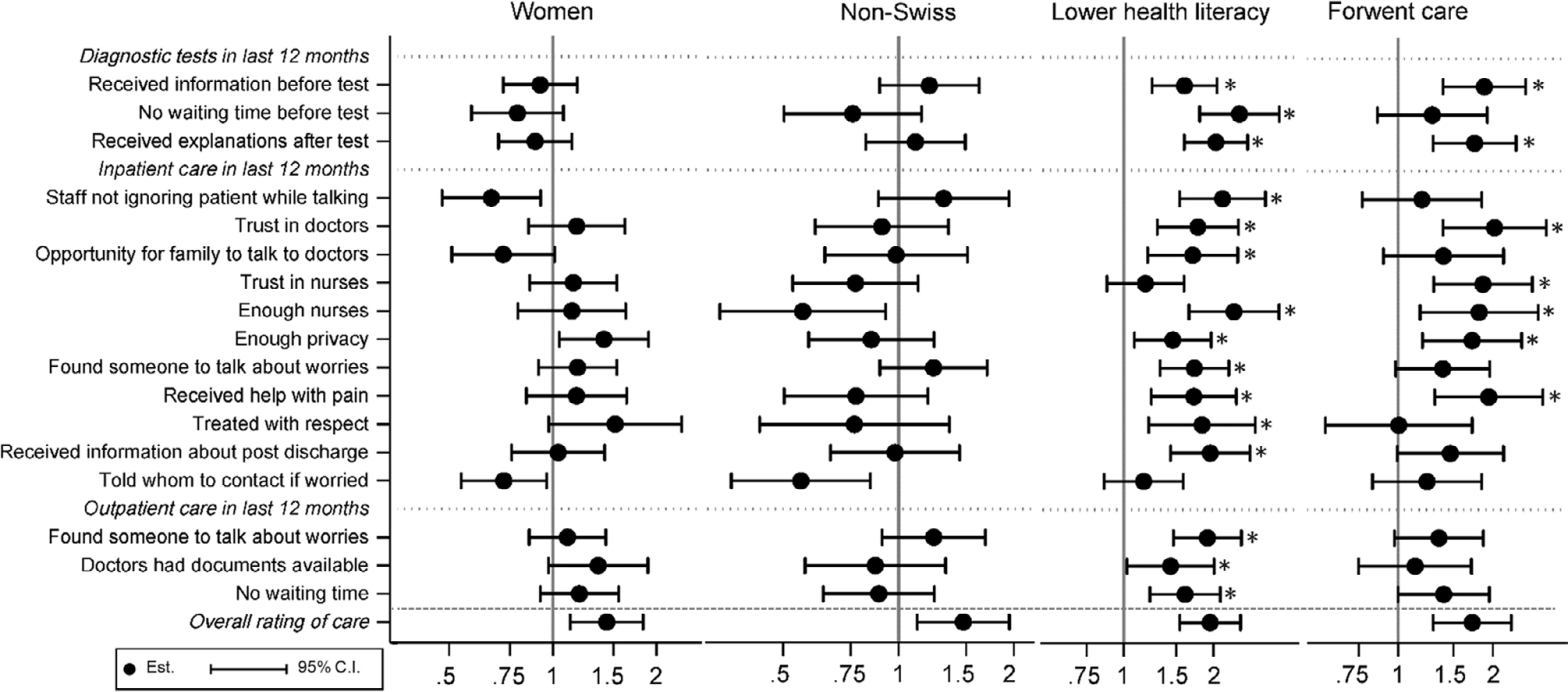
Adjusted OR and 95% CI of patient characteristics (sex, nationality, health literacy, forgoing care) for reporting negative recent experiences of care. * p-value < 0.05 after correction for multiple testing

**Fig. 5:**
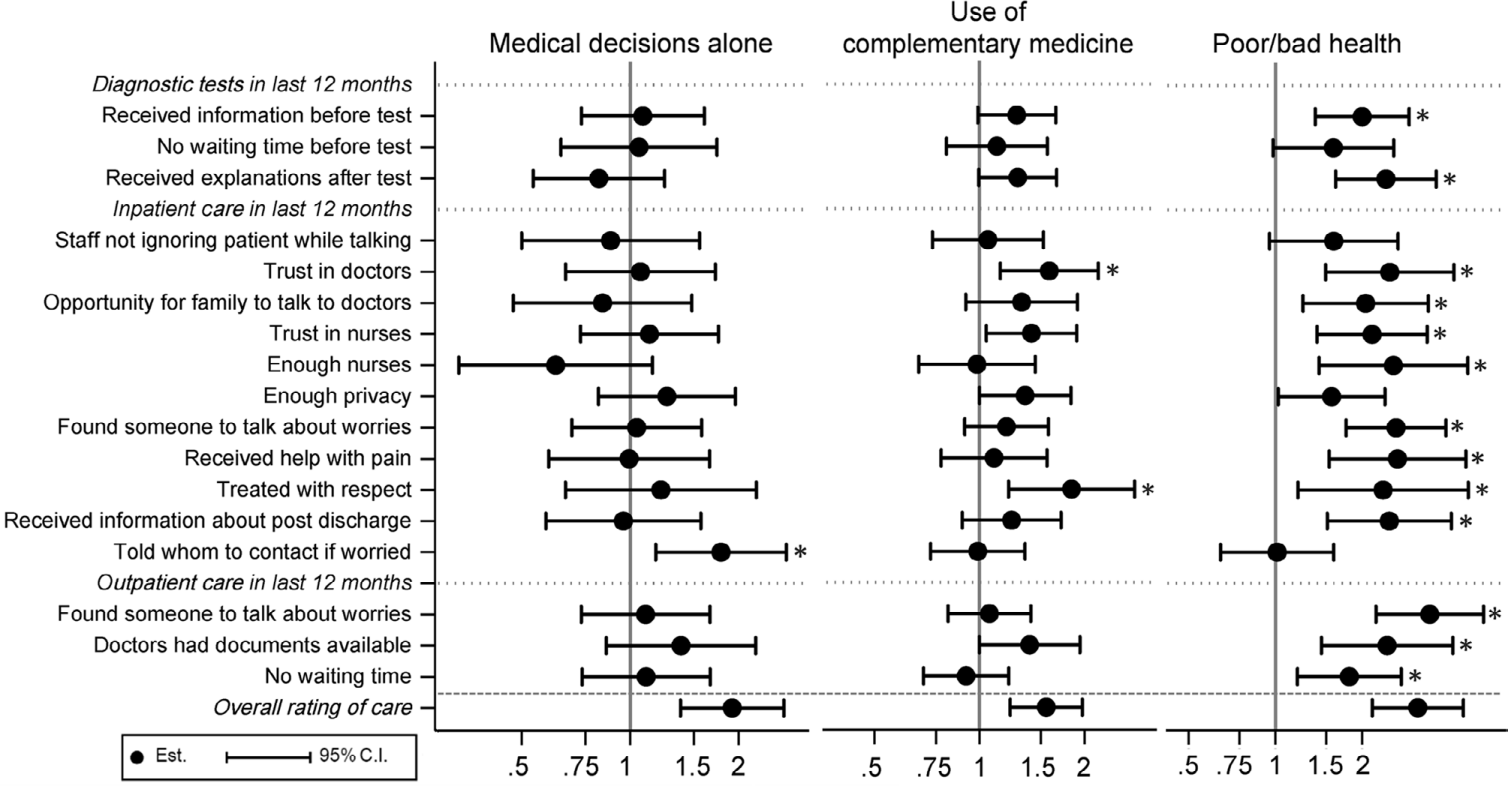
Adjusted OR and 95% CI of patient characteristics (medical decision, complementary medicine, self-reported health) for reporting negative recent experiences of care. * p-value < 0.05 after correction for multiple testing

When examining how these seven factors were associated with the 21 general experiences of care (see Figs. 2 and 3) and the 17 recent experiences of care (see Figs. 4 and 5), three factors were consistently associated with problematic experiences of care: reporting poorer health, having forgone care, and reporting low health literacy. Having used complementary medicine was associated with reporting problematic experiences for about half of the general experiences, especially for experiences during the diagnosis and treatment decision process. These patients were also more likely to not trust doctors and not feel treated with respect. Respondents without the Swiss nationality tended to be more likely to report non-problematic experience, in contrast to their overall rating of care. The gender and decision making preference was not strongly associated with the general and recent experiences of care, in contrast to overall rating.

## Discussion

While rating of overall cancer care was fairly high, it did vary quite substantially across seven patient characteristics: sex, nationality, health literacy, medical decision making preference, financial hardship, health status and use of complementary medicine. Age, education, and marital status were not independent factors, neither were any cancer-related characteristics (e.g., type of cancer, time since diagnosis, treatments received). The variation of experiences of care followed a similar pattern for three of the determinants of overall cancer care (health literacy, financial hardship, health status). Use of complementary medicine tended to also predict problematic experiences of care.

The most important determinant was self-reported health status, where individuals with poor health status were systematically more likely to report a low rating of overall cancer care and problematic experiences of care. This finding concurs with those from previous studies [5, 6, 9, 10, 12, 15, 17–19]. Although our cross-sectional design does not allow to infer the direction of the relationship, other authors have suggested that health status may influence rating of care [17, 18]. One of their explanation is that individuals in poorer health may rate their care more poorly as care is not helping them to improve their health, leading them to have a more negative attitude towards medical care. In a longitudinal study of cancer patients, authors were able to show that a deterioration in global health was linked to a decrease in satisfaction in general, and with doctors in particular [9]. Patients with deteriorating or poor health would have more expectations from care and doctors, which are not fulfilled. Identifying these patients early and providing them with comprehensive support for their health needs could improve their experiences of care, as could discussing their health-related issues. Indeed, one study showed that routine and repeated measurements of quality of life lead to increased discussion of health-related issues, resulting in clinically meaningful improvement in patient well-being [31].

Health literacy was another important determinant in our study, as respondents with low health literacy consistently reported more problematic experiences of care, especially for experiences related to information and explanation around cancer and cancer treatment and related to support. More frequent problems with care reported by patients with low health literacy indicate that having difficulties in understanding medical information may be an important contributor to disparities in care. This evidence adds to the existing evidence that low levels of health literacy in patients are associated with poor health outcomes and inadequate use of health care services (32, 33). Health literacy is a particularly important issue for cancer patients who must navigate a complex and fragmented health care system (32). Limited health literacy was shown to hamper patients’ ability to understand the risks and benefits of cancer treatment [34], which can explain the poor experiences of care reported in our study. Clinicians should pay special attention to providing effective communication and information, to ensure that people with low health literacy have an equal chance to receive the care and support as people with higher health literacy.

Forgoing care due to cost, a proxy of financial hardship, was a strong determinant of lower rating of care and problematic experiences of care. The percentage of patients indicating they forwent care in the last 12 months due to costs was quite high at 13%, a worrying rate in a population of patients who are expected to require regular care and/or follow-ups. The rate was similar to the rate found in a Swiss population-based survey from 2010 and a diabetic population in 2017 [35, 36]. Although Switzerland has universal health insurance coverage, out-of-pocket expenditures is the highest among the country members of the Organisation for Economic Co-operation and Development (OECD) [37], in addition to high health insurance premiums. As these deductible and premiums are independent of income, people with lower incomes pay proportionately more than people with high incomes, which can lead to forgoing care due to costs. Our finding suggest that cancer care in Switzerland is affected by the cost burden put on patients, who reported poor experiences with the current unequitable health system. As forgoing care may lead to worse health status and worse cancer outcomes, health professionals should be aware of this issue and pay attention to patients who might be in this situation, providing them with information on available support.

Complementary and alternative medicine is often used by individuals with cancer to alleviate symptoms, cope with side-effects, and improve physical and emotional well-being [38, 39]. In our sample, 30% of respondents reported having used complementary medicine, similar to the rate reported in the Swiss general population [40]. They were also more likely to report lower overall rating of care and more problematic experiences of care. One hypothesis for this association is provided by previous studies suggesting that patients who were dissatisfied with their medical care were more likely to use complementary medicine [41, 42]. Although the causality cannot be determined from our cross-sectional design, the use of complementary medicine may reflect dissatisfaction and possibly distrust with conventional cancer care, as users were more likely to report poor experiences during the diagnostic process and regarding the handling of treatment side effects. The process of integrating complementary medicine in oncology centers is still beginning in Switzerland, through integrative medicine approaches. Future studies could evaluate whether this negative association between use of complementary medicine and reporting poorer experiences of care reverses in cancer centers offering complementary medicine on site.

In contrast to previous studies, age and education were not independent factors associated with overall rating of care, nor were marital status and living status, our proxies for family support. In addition, none of the cancer-related characteristics (e.g., type of cancer, time since diagnosis, treatments received) were associated with overall cancer care, suggesting that the overall rating of cancer is not determined by the specific cancer trajectory but rather personal characteristics.

The strength of our study resides in the examination of a wide array of potential factors associated with overall rating of care, in a fairly large sample of patients with cancer recruited from four cancer centers in a large region of Switzerland. This was also the first study to assess determinants of patient experiences with cancer care in the French-speaking region of Switzerland. Interpretation of our findings are however limited by several factors. Availability of data was limited to what was collected in the survey. In addition, all data were self-reported, leading to limited information on the specificities of cancer (lack of information on stage at diagnosis for instance) that might be associated with overall rating of care. The cross-sectional nature of the study also prevents drawing conclusions on causality between associated factors and overall rating of care. Regarding the analyses, the decision to dichotomize the main outcome (i.e., overall rating of cancer care) could be seen as a limitation. However, considering the outcome as an interval variable would assume that the scale is perfectly linear, which was not the case as the step shape of the rating distribution shows. It suggests a quite homogeneous majority of people “satisfied” versus the rest of “unsatisfied” people, with a “step” in the distribution located at rating 8. The sensitivity analysis performed with a different cut off at 9 showed similar results, confirming the robustness of the results. The data is also limited by missing data because of skip patterns for the ‘recent’ experiences and where respondents chose or forgot to answer the question. However, imputing missing data using multiple imputation requires a model of the patients’ behavior in order to synthesize data that we do not have and would rely on many assumptions, with a high risk of finding highly biased results.

The patient-related factors associated with ratings of care identified in this study are important information for health professionals. Indeed, patient with those characteristics appear to require additional attention or even specific interventions to ensure that delivery of care is responding to their specific needs and improve their experiences of care. Among these patient groups, those with lower health literacy could benefit from tailored information to ensure that cancer care is explained in a comprehensible way. Patients reporting financial hardship are another group that could benefit from special support to ensure they can obtain the care they need regardless of their ability to pay. The identified determinants are also important information at a policy level and when comparing performance of cancer centers. Indeed, the distribution of the identified patient-related characteristics among patients cared for in cancer centers can have an impact on their global results in patient surveys. Cancer centers serving patients from lower socioeconomic background and with poorer health can advocate for more means to improve the responsiveness of their care.

## Conclusions

Identifying patient-related determinants of patient experiences is useful and valuable to plan efforts for improving patients’ experiences of care and better understand the variability of experiences of care. Among the determinants identified, health literacy and financial hardship emerged as key recurring factors shaping poor patient experiences that should be prioritized for attention by cancer care services to ensure the provision of care meeting all of patients’ needs, including those in poorer health, with lower health literacy, and facing financial hardship.

### Electronic supplementary material

Below is the link to the electronic supplementary material.

Supplementary Material 1

## Data Availability

Data and survey materials are available from the corresponding author upon reasonable request or from data.unisante.ch, the institutional data repository under DDI Document ID 10.16909-DATASET-20.

## Abbreviations

CI: Confidence intervals
FACT-G7: Functional assessment of cancer therapy – General 7 item version
OECD: Organisation for Economic Co-operation and Development
OR: Odds ratio
PREMs: Patient-reported experiences of care measures
SCAPE: Swiss Cancer Patient Experiences
