# Supplementary material for: Socio-demographic and health-related determinants of patients’ overall rating and experiences of cancer care
Source: BMC Cancer. 2023 Sep 29;23:918. doi: 10.1186/s12885-023-11445-6 (PMC10540394; doi:10.1186/s12885-023-11445-6)
Supplement: Supplementary file 1 — Supplementary Material 1 [file 12885_2023_11445_MOESM1_ESM.pdf]

Suppl. Table 1. Sensitivity analyses on the primary analyses with a low rating of care defined as a rating between 0 and 8 (instead of between 0 and 7)

Variable	N (%)	Low rating of overall cancer care n (%)	Crude OR (95% CI)	Adjusted OR* (95% CI)
**Socio-demographic characteristics**				
Sex			p = 0.002	p = 0.019
Men	1046 (39.0)	415 (39.7)	1	1
Women	1634 (61.0)	746 (45.7)	1.28 (1.09-1.50)	1.24 (1.03-1.48)
Age			p < 0.001	-
18-54	591 (22.5)	289 (48.9)	1	
55-64	668 (25.4)	312 (46.7)	0.92 (0.73-1.14)	
65-74	848 (32.2)	338 (39.9)	0.69 (0.56-0.86)	
75+	524 (19.9)	202 (38.6)	0.66 (0.52-0.83)	
Marital status			p = 0.103	-
Married/partnership	1598 (59.8)	673 (42.1)	1	
Separated/divorced	510 (19.1)	228 (44.7)	1.35 (1.05-1.75)	
Single	274 (10.3)	136 (49.6)	1.11 (0.91-1.36)	
Widowed	289 (10.8)	120 (41.5)	0.98 (0.76-1.26)	
Living situation			p = 0.488	-
Living with adult partner	1770 (66.2)	752 (42.5)	1	
Living without adult partner	767 (28.7)	345 (45.0)	1.11 (0.93-1.31)	
Other living arrangements	138 (5.2)	58 (42.0)	0.98 (0.69-1.39)	
Education			P = 0.733	-
Primary	418 (15.9)	188 (45.0)	1	
Secondary	1314 (50.0)	563 (42.9)	0.92 (0.73-1.14)	
Tertiary	898 (34.1)	386 (43.0)	0.92 (0.73-1.17)	
Professional activity status			p = 0.001	-
Active	729 (27.4)	325 (44.6)	1	
Disability or sick leave	287 (10.8)	146 (50.9)	1.29 (0.98-1.69)	
Retired	1395 (52.5)	555 (39.8)	0.82 (0.69-0.98)	
Other	248 (9.3)	121 (48.8)	1.18 (0.89-1.58)	
Principal language			p = 0.180	-
French	2312 (86.3)	991 (42.9)	1	
Other	367 (13.7)	171 (46.6)	1.16 (0.93-1.45)	
Nationality			p = 0.007	p = 0.390
Swiss	2230 (83.3)	219 (49.1)	1	1
Non-Swiss	446 (16.7)	940 (42.2)	1.32 (1.08-1.62)	1.11 (0.88-1.39)
Health literacy (difficulty understanding written medical information)			p < 0.001	p < 0.001
High (never/occasionally)	1905 (72.3)	707 (37.1)	1	1
Low (sometimes/often/always)	731 (27.7)	436 (59.6)	2.50 (2.10-2.98)	2.15 (1.78-2.61)
Preference for making medical decisions			p = 0.001	p < 0.001
With doctor	2239 (84.3)	964 (43.1)	1	1
Alone	249 (9.4)	128 (51.4)	1.40 (1.08-1.82)	1.40 (1.05-1.86)
Doctor	169 (6.4)	57 (33.7)	0.67 (0.48-0.94)	0.52 (0.36-0.75)
Had trouble paying household bills			p = 0.003	-
No	2091 (78.9)	870 (41.6)	1	
Yes	559 (21.1)	272 (48.7)	1.33 (1.10-1.60)	
Forwent care due to costs			p < 0.001	p = 0.004
No	2294 (86.6)	939 (40.9)	1	1
Yes	354 (13.4)	200 (56.5)	1.87 (1.49-235)	1.45 (1.12-1.87)
**Health-related characteristics**				
Type of cancer			p = 0.029	-
Breast	1084 (40.2)	509 (47.0)	1	
Hematological	432 (16.0)	182 (42.1)	0.82 (0.66-1.03)	
Lung	405 (15.0)	113 (40.2)	0.78 (0.62-0.98)	
Colorectal	281 (10.4)	113 (40.2)	0.76 (0.58-0.99)	
Prostate	230 (8.5)	101 (43.9)	0.88 (0.66-1.18)	
Melanoma	138 (5.1)	53 (38.4)	0.70 (0.49-1.01)	
Several	126 (4.7)	43 (34.1)	0.59 (0.40-0.86)	

Type of diagnosis			p = 0.566	-
First cancer	2127 (80.5)	923 (43.4)	1	
Recurrence	271 (10.3)	125 (46.1)	1.12 (0.87-1.44)	
2nd or 3rd cancer	243 (9.2)	101 (41.6)	0.93 (0.71-1.21)	
Time since first treatment			p = 0.625	-
<1 year	729 (27.7)	307 (42.1)	1	
1-5 years	1260 (47.9)	558 (44.3)	1.09 (0.91-1.31)	
>5 years	640 (24.3)	275 (43.0)	1.04 (0.84-1.28)	
Treatment(s) received			p = 0.132	-
Surgery	1626 (28.6)	716 (44.0)	1.06 (0.91-1.24)	
Chemotherapy	1550 (27.3)	665 (42.9)	0.95 (0.81-1.11)	
Radiotherapy	1400 (24.7)	623 (44.5)	1.09 (0.94-1.27)	
Hormonotherapy	748 (13.2)	358 (47.6)	1.28 (1.08-1.52)	
Immunotherapy	352 (6.2)	149 (42.3)	0.95 (0.76-1.19)	
Use of complementary medicine			p < 0.001	p = 0.008
No	1782 (69.4)	739 (41.5)	1	1
Yes	785 (30.6)	381 (48.5)	1.33 (1.12-1.58)	1.29 (1.07-1.55)
Chronic comorbidities			p < 0.001	-
None	1067 (40.7)	414 (38.8)	1	
≥1 other than cancer	1553 (59.3)	725 (46.7)	1.38 (1.18-1.62)	
Overall health status			p < 0.001	p < 0.001
Excellent / Very good	660 (25.0)	172 (26.1)	1	1
Good	1532 (57.9)	717 (46.8)	2.86 (2.04-3.05)	2.23 (1.80-2.75)
Poor/bad	453 (17.1)	261 (57.6)	3.86 (2.99-4.98)	3.03 (2.30-4.01)
Depressive symptoms			p < 0.001	-
No	1678 (63.1)	627 (37.4)	1	
Yes	980 (36.9)	526 (53.7)	1.94 (1.66-2.28)	
Quality of life (0-28 highest)			p < 0.001	-
23-28	653 (24.7)	175 (26.8)	1	
20-22	700 (26.5)	293 (41.9)	1.97 (1.56-4.47)	
17-19	592 (22.4)	282 (47.6)	2.48 (1.96-3.15)	
0-16	701 (26.5)	404 (57.6)	3.72 (2.96-4.67)	

*Adjusted odds ratios from the multivariate model with the seven variables
